# Anti-Inflammatory and Antioxidant Activities of the Methanolic Extract of *Cyrtocarpa procera* Bark Reduces the Severity of Ulcerative Colitis in a Chemically Induced Colitis Model

**DOI:** 10.1155/2020/5062506

**Published:** 2020-04-21

**Authors:** Mario Rodriguez-Canales, Elizdath Martinez-Galero, Alma D. Nava-Torres, Luvia E. Sanchez-Torres, Leticia Garduño-Siciliano, Maria Margarita Canales-Martinez, Luis I. Terrazas, Marco A. Rodriguez-Monroy

**Affiliations:** ^1^Laboratorio de Diabetes y Toxicología de la Reproducción-Teratogénesis, Departamento de Farmacia, Escuela Nacional de Ciencias Biológicas, Instituto Politécnico Nacional, Av. Wilfrido Massieu S/N, Unidad Adolfo López Mateos, Mexico City, Mexico; ^2^Lab. Investigación Biomédica en Productos Naturales, Carrera Medicina, FES Iztacala, UNAM, Avenida de los Barrios Número 1, Colonia Los Reyes Iztacala, Estado de México, C.P, Tlalnepantla, 54090, Mexico; ^3^Departamento de Inmunología, Escuela Nacional de Ciencias Biológicas, Instituto Politécnico Nacional, Prolongación de Carpio y Plan de Ayala s/n, Ciudad de México 11400, Mexico; ^4^Laboratorio de Toxicología de Productos Naturales, Departamento de Farmacia, Escuela Nacional de Ciencias Biológicas, Instituto Politécnico Nacional, Av. Wilfrido Massieu S/N, Unidad Adolfo López Mateos, Mexico City, Mexico; ^5^Lab. Farmacognosia, UBIPRO, FES Iztacala, UNAM, Avenida de los Barrios Número 1, Colonia Los Reyes Iztacala, Estado de México, C.P, Tlalnepantla, 54090, Mexico; ^6^Unidad de Biomedicina, FES Iztacala, UNAM, Avenida de los Barrios Número 1, Colonia Los Reyes Iztacala, Estado de México, C.P, Tlalnepantla, 54090, Mexico

## Abstract

*Cyrtocarpa procera* is a plant used in traditional Mexican medicine to treat different gastrointestinal problems. Here, we investigated the effects of a *C. procera* methanolic extract in DSS-induced colitis mice. Ulcerative colitis (UC) was induced by administering 4% DSS in drinking water to female BALB/c mice. Compared to untreated mice with UC, the treatment group receiving the *C. procera* extract presented less severe UC symptoms of diarrhea, bleeding, and weight loss. Additionally, colon shortening was significantly reduced, and at the microscopic level, only minor damage was observed. Levels of proinflammatory cytokines such as TNF-*α*, IL-1*β*, and IFN*γ* in serum as well as the MPO activity in the colon were significantly reduced in the *C. procera* methanolic extract-treated group. Moreover, the extract of *C. procera* reduced oxidative stress during UC, preventing the deterioration of the activity of antioxidant enzymes such as SOD, CAT, and GPx. Additionally, the extract decreased lipid peroxidation damage and its final products, such as malondialdehyde (MDA). In agreement with this, *in vitro* assays with the *C. procera* extract displayed good antioxidant capacity, probably due to the presence of polyphenolic compounds, in particular the flavonoids that were identified, such as chrysin, naringenin, kaempferol, and catechin, which have been reported to have anti-inflammatory and antioxidant activities. Therefore, the improvement of UC by the *C. procera* methanolic extract may be related to the action mechanisms of these compounds.

## 1. Introduction

Ulcerative colitis (UC) is an inflammatory disease limited to the colonic mucosa [1, 2] that is characterized by a variety of symptoms, including abdominal pain and cramping, bloody diarrhea, rectal bleeding, weight loss, fever, and fatigue, which may begin gradually or start all at once [3, 4]. Currently, the pathogenesis of UC remains unknown, but it has been related to multifactorial mechanisms involving interactions between genetic and immunological and environmental factors [5]. Nevertheless, there is no evidence that any of these factors are the direct cause of UC, which means that the etiology of the UC remains unclear [6].

This disease is considered a problem in modern society due to its high incidence, which has increased since the last decade [1, 7]. In fact, the incidence and prevalence of inflammatory bowel diseases, where UC is the principal disease together with Crohn's disease, is increasing in Northern Europe, the United Kingdom, and the United States [8], and even in regions where its incidence has been considered low, including Latin America [7].

One of the most commonly used drugs for UC is 5-aminosalicylic acid (5-ASA). It has been demonstrated that 5-ASA has a good safety profile and high effectiveness against UC. Nevertheless, 5-ASA is not exempt from side effects such as headache, nausea, flatulence, and diarrhea. Other rare but serious side effects include pleuritic pericarditis, myocarditis, pancreatitis, and cholestatic hepatitis [9]. Therefore, there is a need to search for new therapeutic options that are effective and have fewer side effects. Thus, natural products and their chemical compounds have been proposed as candidates for the development of new drugs due to their broad therapeutic spectrum, low toxicity, low occurrence of side effects, and low cost [10].

*Cyrtocarpa procera* Kunth, commonly known as “chupandilla,” is a plant endemic to Mexico, distributed mainly in the states of Jalisco, Michoacan, Nayarit, Guerrero, Mexico, Morelos, Puebla, and Oaxaca. Its bark is used in Mexican traditional medicine [11] to treat different diseases, including dysentery and diarrhea [12, 13]. It has been shown that *C. procera* reduces the severity of gastritis induced by ethanol [14]. In addition, it is worth mentioning that the bark of *C. procera* is used as an adulterant of the bark of *Amphipterygium adstringens* (cuachalalate) because they look very similar [15].

*A. adstringens* is also used in Mexican traditional medicine to treat different gastrointestinal diseases, such as gastritis, gastric ulcers, and gastrointestinal cancer [4, 13]. Moreover, our recent work demonstrated that the methanolic extract of the bark of *A. adstringens* decreases UC severity by reducing both inflammation and oxidative stress [4], two of the most important factors in UC establishment and progression [16].

Therefore, we decided to evaluate the antioxidant and anti-inflammatory effects of the methanolic extract of *C. procera* in a mouse model of colitis induced by dextran sulfate sodium (DSS), performing determinations of antioxidant enzymes and proinflammatory cytokines in addition to macroscopic and microscopic evaluations of the severity of UC and a chemical characterization of the methanolic extract of *C. procera*.

## 2. Materials and Methods

### 2.1. Reagents

DSS (MW: 35,000–50,000; MP Biomedicals, Solon, OH, USA) was used for the induction of colitis. SOD, CAT, GPx, and TBARS kits were obtained from Cayman Chemical (Ann Arbor, MI, USA). An OxySelect myeloperoxidase activity assay kit was obtained from Cell Biolabs Inc. (San Diego, CA, USA) and was used to determine the colonic antioxidant enzymatic activities. Determination of cytokine levels was performed using a Bio-Plex Pro Mouse Cytokine 8- Plex Panel (Bio-Rad, Hercules, CA, USA). For antioxidant activity, 2,2-diphenyl-1-picrylhydrazyl solution (DPPH) (Sigma-Aldrich, cat. D913-2) was used. For total phenolic quantification, Folin-Ciocalteu's reagent (Hycel, HYC-2790-250) was used. The concentration of total flavonoid content was determined using aluminum chloride (Fermont, PQ-24011).

### 2.2. Plant Material

*C. procera* bark was collected in June 2017 in the Tehuacan-Cuicatlan biosphere reserve region, located between the states of Oaxaca and Puebla. In this region, medicinal uses of both *C. procera* and *A. adstringens* barks have been reported for the treatment of gastrointestinal diseases, as mentioned above [13, 14]. *C. procera* specimens were collected in the field with permission from the “Secretaria de Medio Ambiente y Recursos Naturales” (SGPA/DGVS/1266) and botanically identified by M.C Maria Edith Lopez Villafranco (curator of the IZTA Herbarium). A voucher specimen was deposited in the IZTA Herbarium at the Facultad de Estudios Superiores Iztacala, UNAM (voucher number IZTA-2412).

### 2.3. Mice

Female BALB/c mice 6-7 weeks of age (19 g ± 1 g of body weight) were used. They were maintained in a pathogen-free environment, at the FES Iztacala, UNAM animal care facility, according to the Faculty Animal Care and Use Committee and government guidelines (official Mexican regulation NOM-062-ZOO-1999), in strict accordance with recommendations in the Guide for the Care and Use of Laboratory Animals of the National Institutes of Health (USA). Animals were acclimatized for a week before the experiments. They maintained in cages with wood shavings and bedding in the animal house (23°C ± 2°C ambient temperature and 12 h light-dark cycles). They had access to food pellets and water ad libitum during the whole experiment. For all tests, animals were randomly assigned to each experimental group by giving them numbers and picking aleatory using the Excel software. On each test, the experimental unit was an individual mouse.

Experimental mice protocol was performed in compliance with the Animal and Ethics Review Committee of the Facultad de Estudios Superiores Iztacala UNAM, who approved the protocol under the number CE/FESI/092017/1206 and CE/FESI/012020/1345.

### 2.4. *C. procera* Methanolic Extract

The extract of *C. procera* was obtained from dehydrated bark (973 g) through maceration with methanol (2.0 L) at room temperature. After filtration, the solvent was evaporated under reduced pressure, generating the methanol extract. The yield of the bark was 29% (282.26 g).

### 2.5. Acute Toxicity of the *C. procera* Methanolic Extract

To evaluate the toxicity of the extract, an acute toxicity assay was performed as described in protocol 423 by the OECD [17]. In summary, six female BALB/c mice weighing 25 g were used. Before the administration of the extract, the mice were fasted for four hours. The extract was administered through an orogastric tube at a dose of 2,000 mg/kg body weight, and food was withheld for an additional 2 hours.

After dosing, mice were individually observed during the first 30 min and for the next four hours. Subsequently, observations were made daily for 14 days. Observation and registering of toxicity signs included changes in weight, skin, fur, eyes, mucosal membranes, respiratory system, nervous system, motor activity, and behavior. Signs such as shaking, convulsion, salivation, diarrhea, diuresis, lethargy, sleep, coma, anesthesia, piloerection, and irritability were monitored during the experiment [17].

Animals were weighed twice a week until the end of the study, on day 14. After the mice were sacrificed, a gross necropsy was undertaken, and the brains, hearts, lungs, kidneys, adrenal glands, stomachs, and spleens were observed for the presence of injury or damage or any sign of toxicity.

### 2.6. Experimental Colitis

Colitis was induced through the administration of 4% DSS in drinking water. Briefly, after one week of adaptation, mice were randomly assigned (*n* = 6) to the following experimental groups: Control (untreated; no DSS), DSS (drinking 4% DSS plus 100 *μ*L of saline solution, daily cannulated), and DSS+extract (drinking 4% DSS plus 100 *μ*L of the methanolic extract of *C. procera*, daily cannulated). Doses of the extract were 200 mg/kg mouse body weight.

Losses in weight, stool consistency, and blood were determined daily. These parameters were scored to determine the disease activity index (DAI). A score was assigned depending on the severity as follows: weight change (0: <1%, 1: 1–5%, 2: 5–10%, 3: 10–15%, or 4: >15%), blood (0: negative, 2: positive, or 4: gross bleeding), and stool consistency (0: normal, 2: loose stool, or 4: diarrhea). When the DAI score was the maximum (twelve), on day 10, mice were euthanized as humanly as possible, using a CO_2_ chamber. Blood was collected by cardiac puncture (≈700 *μ*L per mouse) and centrifuged to isolate serum. Immediately after, serum was kept at -20°C until use.

### 2.7. Histology and Histopathological Analysis

After euthanasia and collection of blood samples, the ventral side of the mice was exposed, and an incision was made to access the abdominal cavity. A precise cut was made in the base of the cecum and in the base of the anus. The intestine was gently cleaned with cold PBS and then measured with an electronic Vernier caliper.

For the histological analysis, colon tissue was fixed in 10% formalin and embedded in paraffin. Tissue samples (5 *μ*m thick) were prepared and stained by the conventional method of hematoxylin and eosin (H&E).

Histopathological analysis was performed by and examiner without prior knowledge of the experimental procedures. A score was assigned due to the severity of the parameters analyzed: leucocyte infiltration (0: none; 1: slight; 2: moderate; 3: severe), crypt damage (0: none; 1: basal third damaged; 2: basal two-thirds damaged; 3: loss of entire crypt and epithelium), and extent of injury (0: none; 1: mucosa layer; 2: submucosa layer; 3: muscle layer).

### 2.8. Measurement of Proinflammatory Cytokines

Levels of serum IL-1*β*, TNF-*α*, and IFN*γ* were determined using a Bio-Plex Pro Mouse Cytokine 8-Plex Panel according to the manufacturer's protocol.

### 2.9. Measurement of Antioxidant Enzymatic Activity

The enzymatic activities of superoxide dismutase (SOD), catalase (CAT), and glutathione peroxidase (GPx) were measured in colon tissue samples. After colon tissues were washed with saline solution, a sample from the distant colon (2 cm in length) was taken and homogenized in phosphate buffer using a Bullet Blender (Next Advance, NY, USA). Homogenates were centrifuged (10,000 × g, 15 min, 4°C), and the supernatants were collected for the tests.

The enzymatic activities of SOD, CAT, and GPx were determined using the methods provided by the assay kits (Cayman Chemical Company,) as described previously.

### 2.10. Measurement of Myeloperoxidase Activity

MPO activity was determined in intestinal tissue homogenates using the OxySelect™ myeloperoxidase activity assay kit (Cell Biolabs, Inc. San Diego, CA, USA). The tissue samples were washed with cold sterile 1X PBS prior to homogenization to eliminate MPO from the blood. Approximately, 100 mg of tissue in 1-2 mL of cold 1X PBS, pH 6.0 containing 0.5% HTAB (5% hexadecyltrimethylammonium bromide in PBS) were homogenized using a Bullet Blender homogenizer (Next Advance, NY, USA), and the homogenates were centrifuged at 10,000 × g for 15 min at 4°C. The supernatant was collected for the determination of MPO activity.

### 2.11. Lipid Peroxidation Determined by the Measurement of TBARS

Colon tissue samples (~100 mg each) were homogenized in 250 *μ*L of RIPA buffer (25 mM Tris-HCl pH 7.6, 150 mM NaCl, 1% NP-40, 1% deoxycholic acid, 0.1% sodium dodecyl sulfate) on ice. The homogenates were centrifuged at 10,000 × g for 10 min at 4°C, and the supernatants were subjected to assays.

### 2.12. Antioxidant Activity of the Methanolic Extract of *C. procera*

The antioxidant activity of the methanolic extract of *C. procera* was determined by the DPPH assay, as described previously by Okusa et al. [18]. The electron-donating capacity of the extract was calculated from the bleaching of the purple-colored DPPH solution dissolved in methanol. Ninety-six-well ELISA plates were filled with extract concentrations ranging from 1 to 100 *μ*g/mL and 100 *μ*M DPPH solution [4]. Quercetin was used as a control, and the same concentrations of the extract were used. After 30 min of incubation in a dark room at 37°C, the absorbance was determined at 517 nm in an ELISA SLT Spectra.

The antioxidant activity values were determined according to the following equation:

(1) $$\begin{array}{c} {\text{SC}}_{50}=\left[\left(\text{absorbance of control}-\text{absorbance of sample}\right)/\text{absorbance of control}\right]\times 100. \end{array}$$

The concentration leading to 50% inhibition (SC_50_) was determined graphically.

### 2.13. Total Phenolic and Flavonoid Content

The concentration of total phenolic content in the extract was evaluated using Folin-Ciocalteu's reagent, as mentioned by Das et al. [19].

The concentration of total flavonoid content in the extract was determined using the aluminum chloride colorimetric method described previously by Ramamoorthy [20].

### 2.14. Chemical Composition Analysis by HPLC-MS

The chromatographic separation was accomplished using a HPLC (Infinity Series 1200, Agilent Technologies, Germany) equipped with a Kinetex 2.6 u, C18 100 Å column (150 × 2.1 mm) (Phenomenex, USA). The column temperature was maintained at 25°C. The following gradient program was used, along with a mobile phase consisting of water : aceto-nitrile (90 : 10) with 0.1% formic acid (solvent A) and methanol : acetonitrile (9 : 10) with 0.1% formic acid (solvent B). This initial term for 3 min in an isocratic elution is composed of 100% solvent A followed by 3–11 min: 65% A-35% B; 11–20 min: 55% A-45% B; 20–35 min: 100% B; and 25 min: 100% B, *v*/*v*. The flow rate was 0.2 mL/min, and the injection volume of the *C. procera* methanol extract was 20 *μ*L (3 mg/mL).

HPLC- MS analysis was performed using an Agilent 1200 Infinity LC coupled to an Agilent 6230 TOF with an Agilent Dual ESI Source (ESI SG14289023) and Mass Hunter Wokstation Software, Version B.05.01, Build 5.01.5125.3 operating in the negative ionization mode. Capillary voltage was 4000 V; dry gas temperature was 250°C; nitrogen was used as the dry gas at a flow rate 6 L/min; nebulizer pressure was 60 psi; fragmentor was 200 V; MS range was 50–1300𝑚/𝑧; MS acquisition rate was 1 spectrum/s.

The standards used were vanillin, baicalein, myricetin, genistein, acacetin, luteolin, naringenin, kaempferol, chrysin, pinocembrin, catechin, catechol, naringin, quercetin, caffeine, and apigenin.

## 3. Results

### 3.1. The Methanolic Extract of *C*. *procera* Showed no Acute Oral Toxicity

First, we determined the possible toxicity of our methanolic extract of *C. procera* on young adult mice. In the acute toxicity study, the methanolic extract of *C. procera* at a dose of 2000 mg/kg, which is the highest dose recommended by the OECD in a standard study, did not produce any mortality or signs of toxicity over the observation period. Mice receiving the methanolic extract of *C. procera* for 14 days did not show any signs of toxicity (e.g., change in total weight or behavior), nor were any pathological findings observed in the organs during the necropsy of mice after euthanasia (data not shown). Thus, the oral LD_50_ of the extract in female BALB/c mice is higher than 2000 mg/kg b.w., and according to this protocol, the extract can be classified in the less severe or “unclassified” category.

### 3.2. The Methanolic Extract of *C. procera* Increased the Survival Rate during DSS-Induced Colitis

**Figure 1:**
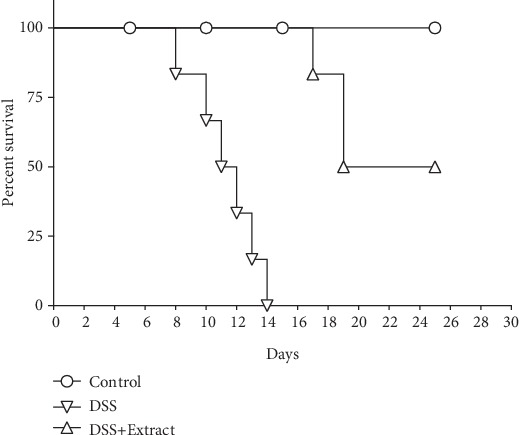
Effects of the methanolic extract of *C. procera* on the % survival during DSS-induced colitis. Experimental groups were formed with an *n* = 6; treatment with the extract was at a doss of 200 mg/kg. It was determined as a human endpoint when each mouse reached the maximum values according to the DAI, that is, the maximum value of weight loss, diarrhea, and bleeding. The experiment was performed in triplicate showing that there is a significant difference between the curves. *P* < 0.0001 versus DSS (Mantel-Cox test).

As expected, the DSS group showed weight loss of over 15%, diarrhea and bleeding due to the severity of colitis; after 14 days of exposure to DSS, 100% of the animals succumbed to colitis. In contrast, the DSS+Extract group displayed less weight loss and the survival rate improved to 50% until the end of the experiment (day 25) after DSS exposure (Figure 1).

### 3.3. Administration of Methanolic Extract of *C. procera* Reduced the Severity of DSS-Induced Colitis

**Figure 2:**
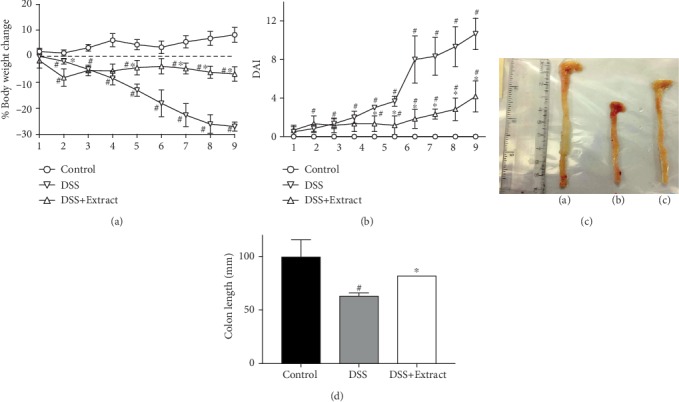
Effect of the methanolic extract of *C. procera* in DSS-induced colitis. (a) % body weight change compared with the initial body weight. (b) Disease activity index, considered as the summary of the % body weight loss, bleeding and diarrhea. (c) Gross picture of colons in the following groups: a) Control, b) DSS, and c) DSS+Extract. (d) Variations in colon length. Data presented are the mean values from all three groups (*n* = 6) ∗*P* < 0.001 vs DSS group, #*P* < 0.001 vs Control group.

Weight loss was present in both groups administered with DSS. Nevertheless, starting on day 5, this loss increased significantly in the DSS group and continued until the end of the experiment, while in the DSS+Extract group, the weight loss was less severe (Figure 2(a)). Along with weight loss, the DSS+Extract group showed slight rectal bleeding and some loss of stool consistency, while the DSS group presented heavy bleeding and diarrhea starting on day 7 onwards. The DAI, which is calculated as a score that includes body weight loss, diarrhea or stool consistency and bleeding, was determined daily. DSS (4%) administration has been associated with significant clinical changes, including weight loss, presence of rectal bleeding and diarrhea, in mice [21]. However, mice treated with methanolic extract of *C. procera* displayed a reduced severity of the disease, with DAI scores that were remarkably lower compared to those of the DSS group (Figure 2(b)).

Another characteristic that has been described as a marker of colitis severity in experimental models is colon shortening [22]. The DSS+Extract group (81.59 ± 6.55 mm) prevented the shortening present in the DSS group (62.84 ± 3.08 mm); interestingly, the DSS+Extract group was not significantly different from the Control group (99.35 ± 16.15 mm) (Figures 2(c) and 2(d)), suggesting a protective role for the methanolic extract of *C. procera* in colitis development.

### 3.4. Acute Administration of the Methanolic Extract of *C. procera* Reduced the Histopathological Score

**Figure 3:**
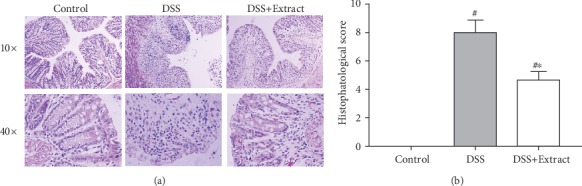
(a) Representative paraffin sections of colonic tissue from each group by hematoxylin and eosin staining. Observations were made under a microscope at 10x and 40x. (b) Histopathological score. The data shown are the mean values for each group (*n* = 6). ∗represents significant differences vs DSS group (*P* < 0.05) and #*P* < 0.001 vs Control group (one-way ANOVA).

It is well known that DSS-induced colitis in mice leads to the destruction of the crypt structure, alteration of the epithelial layer, and massive infiltration of inflammatory cells in the colonic tissue when compared to its normal morphology [21]. As expected, the Control group did not present any signs of damage or infiltration of inflammatory cells, while the DSS group had severe crypt damage and a loss of structure. A large number of inflammatory cells were present in the mucosa and submucosa layers. Consistent with the macroscopic results obtained, the DSS+Extract group showed protection against loss of the architecture, and inflammatory cells were less abundant in the mucosa and submucosa layers (Figure 3).

### 3.5. The Methanolic Extract of *C. procera* Reduced Proinflammatory Cytokine Levels in Ulcerative Colitis

**Figure 4:**
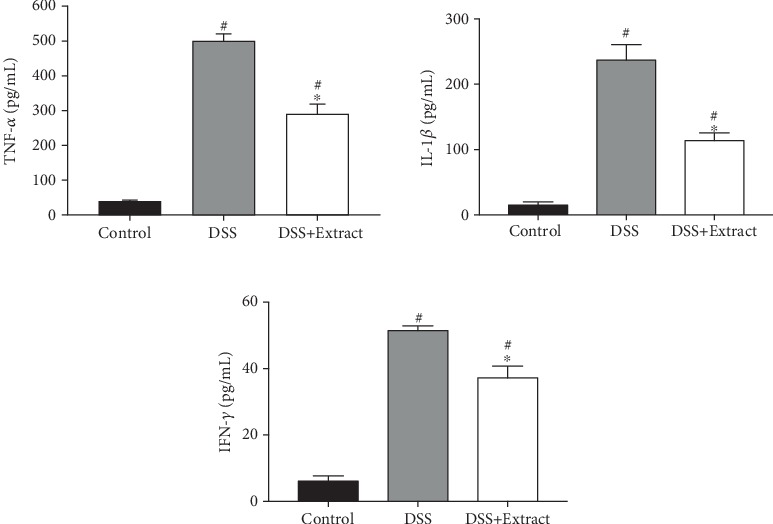
Effect of the *C. procera* methanolic extract on TNF*α*, IL-1*β*, and IFN*γ* serum levels. Data presented are mean values from all three groups (*n* = 6). ∗represents significant differences vs DSS group (*P* < 0.0001) and #*P* < 0.0001 vs Control group (one-way ANOVA).

Elevated levels of TNF-*α*, IL-1*β*, and IFN*γ* due to activation of immune cells are hallmarks of colitis, in both humans and mice. Even though both groups of mice exposed to DSS had increased levels of these three cytokines, it was found that the group treated with the methanolic extract of *C. procera* showed statistically lower levels compared to the DSS group (*P* < 0.001), which means that the *C. procera* extract had a preventive effect against exacerbated inflammatory cytokine production (Figure 4).

### 3.6. The *C. procera* Extract Reduces MPO Activity Levels in Colon Tissue

**Figure 5:**
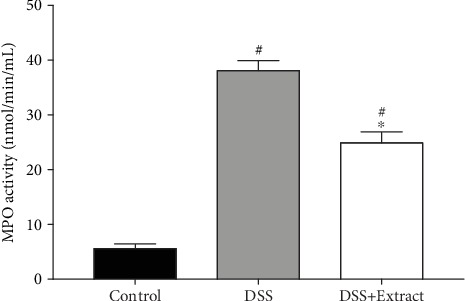
MPO activity in colonic tissue. Colons were resected after 10 days, processed, and the supernatants were recovered to measure the MPO activity. The data shown are the mean values for each group (*n* = 6). ∗represents significant differences vs DSS group (*P* < 0.0001) and vs Control group (one-way ANOVA).

MPO is an enzyme present almost exclusively in neutrophils; thus, MPO activity might be proportional to the number of neutrophils recruited to the inflamed colon. Accordingly, the administration of the *C. procera* methanolic extract significantly reduced MPO activity levels during DSS-induced colitis, which indicates that fewer neutrophils infiltrated the mucosa and submucosa compared with the DSS group (Figure 5).

### 3.7. The *C. procera* Extract Decreased Oxidative Stress in DSS-Induced Colitis

As mentioned earlier, oxidative stress is considered a key factor in colitis progression and severity [16]. We found that the *C. procera* methanolic extract contains polyphenolic compounds, among which flavonoids can be highlighted due to their high antioxidant activity [23]. In agreement with these results, the extract has good antioxidant potential (Table 1) in the DPPH reduction assay, presenting an antioxidant capacity (AC_50_) of 34.44 *μ*g/mL, while quercetin, used as a standard, showed an AC_50_ of 16.34 *μ*g/mL (Table 1).

**Figure 6:**
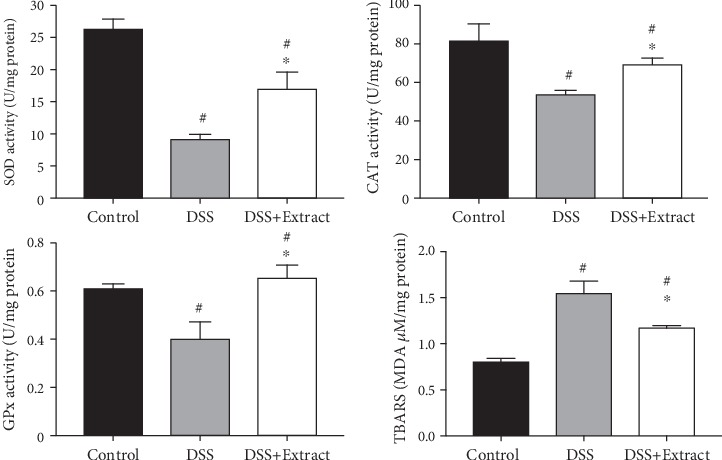
Effects of the *C. procera* methanolic extract on the enzymatic activity of SOD, CAT, and GPx, and the MDA levels during DSS-induced colitis. Determinations were made in homogenized colon tissue. The data are presented as the mean values per group (*n* = 6). ∗represents significant differences vs DSS group (*P* < 0.0001) and #*P* < 0.0001 vs Control group (one-way ANOVA).

Exposure to DSS has been shown to cause a high production of free radicals that cause oxidative stress. The antioxidant enzymes SOD, CAT, and GPx play not only fundamental but also indispensable roles in the antioxidant protective capacity of biological systems against free radical attack [24]. Importantly, the *C. procera* methanolic extract reversed the loss of SOD, CAT, and GPx enzymatic activity compared to the DSS group (Figure 6).

Furthermore, the *C. procera* extract decreased the levels of MDA, a final product of lipid peroxidation, which is considered a hallmark of cellular damage as a result of oxidative stress [25]. Together, these results showed that the administration of *C. procera* improves antioxidant defenses, which prevents oxidative injury, lipid peroxidation, and the ensuing damage produced by this process, such as damage to membranes and the impairment of cellular proteins and nucleic acids (Figure 6).

### 3.8. Chemical Composition Analysis of the *C. procera* Methanolic Extract according to HPLC

The compounds in the methanolic extract were identified by comparing the retention times and absorption spectra of flavonoid standards. It was possible to identify chrysin, naringenin, kaempferol, catechin, and quercetin as part of the composition of the *C. procer*a methanolic extract. A bibliographic study of the anti-inflammatory or antioxidant activities of these compounds is shown in Table 2.

## 4. Discussion

UC is a chronic inflammatory bowel disease, and its incidence worldwide has been increasing over the years [33]. Currently, traditional treatments for UC include aminosalicylates (sulfasalazine or mesalazine) and immunosuppressants (glucocorticoids, azathioprine, and methotrexate), which can produce adverse effects when used over the long term [23]. Natural products and their derivatives have been proposed as a source for new drug research and development due to their broad spectrum of therapeutic effects and their less severe side effects [10, 34].

Here, we tested *C. procera*, a medicinal plant used in Mexican traditional medicine for gastrointestinal diseases, in a DSS-induced mouse colitis model, which is a useful model for screening anti-inflammatory and antioxidant effects due to its high similarity with human UC in the production of proinflammatory cytokines and reactive oxygen species (ROS) [35].

First, we found that mice treated with high doses of the *C. procera* methanolic extract did not show any signs of toxicity; thus, this *C. procera* extract is in category 5 or unclassified (the lowest toxicity category) according to OECD procedures [17]. This result corroborates the safety of *C. procera* bark extracts since in previous studies that administered doses of 5000 mg/kg, it was reported that the different extracts of *C. procera* were not toxic at this dose [36], confirming that its use is safe.

Next, our results obtained in this investigation showed that the administration of the *C. procera* methanolic extract reduces the severity of DSS-induced colitis; this statement is supported by a notable increase in the survival rate that was associated with a significant decrease in several signs of the disease, such as diarrhea, bleeding, and weight loss severity, which were reflected in lower DAI score values. Additionally, the inhibition of colon shortening was lower when the *C. procera* extract was administered to colitic mice.

According to these results, the microscopic analysis revealed that *C. procera* extract treatment decreased damage to colonic structures and inflammatory infiltration in the intestinal mucosa and submucosa layers compared with the DSS group.

This improvement in colitic mice receiving the *C. procera* methanolic extract was associated with a significant inhibition of systemic levels of proinflammatory cytokines, including TNF-*α*, which plays a central role in UC because its signaling produces different proinflammatory effects, such as activation of NF-*κ*B, augmented angiogenesis, and activation of macrophages and effector T cells [37], which results in severe intestinal tissue damage.

Another well-known marker of UC severity is MPO activity, which is directly related to neutrophil infiltration in the colonic mucosa and submucosa [38]. Here, we found that the early administration of the *C. procera* extract significantly decreased MPO activity, supporting the idea that the *C. procera* extract has anti-inflammatory properties.

Increased inflammation in colon tissue results in overproduction of ROS and the generation of oxidative stress, which, in turn, generates more damage to intestinal cells and promotes more inflammation [39]. Importantly, during UC, ROS can also generate DNA damage and, ultimately, carcinogenesis at the site.

SOD, CAT, and GPx are part of the enzymatic antioxidant defense against ROS damage. Their function, as the first line of defense antioxidants, is related to the fast suppression or prevention of the formation of free radicals or ROS. It has been described that during UC, the enzymatic activity of SOD, CAT, and GPx is reduced [24]. In this study, we demonstrated that the *in vivo* administration of the *C. procera* methanolic extract enhances SOD, CAT, and GPx activity in colonic tissue compared with the DSS group, contributing to the overall decrease in the severity of DSS-induced colitis. Furthermore, the extract showed good antioxidant capacity on the DPPH *in vitro* assay.

The extract of *C. procera* showed a chemical composition rich in phenolic compounds. A growing body of evidence confirms that phenols, particularly flavonoids, exhibit potent anti-inflammatory and antioxidant activities, both *in vitro* and *in vivo* [40]. In addition, it has been reported that treatment with different flavonoids or their glycosides significantly reduces colonic mucosal injury during UC in TNBS-, acetic acid-, and DSS-induced colitis experimental models. This can be explained through different mechanisms, including a reduction in and protection against oxidative stress, preservation of epithelial barrier function, a reduction immune cell responses, and correction of the dysbiosis in the gut microbiota [40].

To better understand the chemical composition of the methanolic extract of *C. procera*, an HPLC analysis was performed to determine flavonoids. Chrysin, naringenin, kaempferol, catechin, and quercetin were the compounds that could be identified. As mentioned before (Table 2), it has been shown that all of these compounds exhibit, among others, antioxidant or anti-inflammatory activities [27]. Chrysin is also capable of diminishing inflammatory markers such as MPO activity, TNF-*α* levels and NF-*κ*B activation and translocation to the nucleus if administered previously to DSS-induced colitis mice [26]. In an *in vitro* assay, Caco-2 cells exposed to the proinflammatory cytokine IL-1*β* favor the production of PGE2, but when the cells were costimulated with chrysin, such mediators of inflammation were inhibited, and this effect was attributed to a reduction of prostaglandins derived from COX-2 activity [41]. In acetic acid-induced colitis, naringenin, another flavonoid found in our extract, was capable of reducing colonic inflammation by preventing mucus depletion, decreasing levels of inflammatory cytokines such as TNF-*α*, IL-6, and IL-1*β*, and downregulating the expression of genes such as COX-2 and iNOS. Naringenin has also shown antioxidant activity by preventing the impaired activity of SOD and CAT during UC. Naringenin also decreases LPO-specific final products such as MDA [28].

Catechin and quercetin are two important flavonoids present in the current extract. Both have been demonstrated to alleviate colonic inflammation by reducing T cell infiltration to the gut and proinflammatory cytokine production expression, in addition to their antioxidant activities. All these data suggest that the beneficial effects of the *C. procera* methanolic extract on DSS-induced colitis could be explained, at least in part, by some of the polyphenolic compounds present in the bark, particularly the flavonoids.

Because *A. adstringens* and *C. procera* are members of the same family, Anacardiaceae, and due to their commercial presentation, these, like many other plants sold in markets, are liable to adulteration [14]. In this work, we demonstrated that the biomedical and therapeutic properties that the *C. procera* methanolic extract exerted in this DSS-induced colitis model are very similar to those of *A. adstringens*, which was studied previously by our work team [4]. Both *C.* procera and *A. adstringens* extracts had remarkable antioxidant and anti-inflammatory effects and greatly diminished UC severity. Even in their chemical composition, they were very similar regarding the phenols and flavonoids present, and although other types of chemical compounds have been identified in other studies, there have been differences between these species [14]. All the above reasons explain why *C. procera* bark is used in the market to adulterate that of *A. adstringens* bark, and why people consume their mixture without distinction.

## 5. Conclusions

In recent studies, it has been suggested that the administration of antioxidants, with additional anti-inflammatory effects, may be beneficial for the treatment of UC. We demonstrated that *C. procera*, a medicinal plant used in Mexican traditional medicine, induces different beneficial effects on DSS-induced colitis, such as an improvement of UC severity and the suppression of inflammatory enhancement of antioxidant defenses, and by thus, the reduction of oxidative injuries and lipid peroxidation damage. Therefore, the practical nontoxicity of the plant extract, together with the pharmacological properties shown here, encourages further study of the plant; although, it will be necessary to continue with more safety studies to validate its clinical use.

## Figures and Tables

**Table 1:** Total phenolic and total flavonoid contents and antioxidant capacity of the *C. procera* methanolic extract.

	TPC-GAe	TFC-Qe	AC_50_
*C. procera* methanolic extract	360 mg/g	3.6 mg/g	34.44 *μ*g/mL
Quercetin	—	—	16.34 *μ*g/mL

The total phenolic content is reported as gallic acid equivalents (TPC-GAe), and the total flavonoid content is reported as quercetin equivalents (TFC-Qe) per gram of dry extract.

**Table 2:** Flavonoids identified as part of the chemical composition of the *C. procera* methanolic extract.

Flavonoid	Retention time (min)	Parent ion (m/z) [M-H]^−^	Relative error (ppm)	Anti-inflammatory activity	Antioxidant activity	Reference
Chrysin	37.440	253.0478	0.51	+	+	[26, 27]
Naringenin	29.354	271.0583	0.85	+	+	[28]
Kaempferol	33.846	285.0365	-0.23	+	+	[29, 30]
Catechin	6.820	289.0700	-3.16	+	+	[31]
Quercetin	17.048	301.0332	0.07	+	+	[32]

## Data Availability

The data used to support the findings of this study are available from the corresponding author upon request.

## Supplementary Materials

Supplementary Materials Reporting in vivo experiments, in which we point out the points that our study meets with the NC3Rs initiative to improve the design, analysis, and publication in animal research. The photographic record of the toxicity assessment of the extract. This toxicity assay was performed as described in protocol 423 by the OECD. Pathological findings not were observed in the organs during the necropsy of mice after euthanasia. Before the administration of the extract, the mice were fasted for four hours. The extract was administered through an orogastric tube at a dose of 2,000 mg/kg body weight, and food was withheld for an additional 2 hours. Mice receiving the methanolic extract of C. procera for 14 days did not show any signs of toxicity, nor were any pathological findings observed in the organs during the necropsy of mice after euthanasia.
