# Supplementary material for: Anti-Inflammatory and Antioxidant Activities of the Methanolic Extract of Cyrtocarpa procera Bark Reduces the Severity of Ulcerative Colitis in a Chemically Induced Colitis Model
Source: Mediators Inflamm. 2020 Apr 21;2020:5062506. doi: 10.1155/2020/5062506 (PMC7191396; doi:10.1155/2020/5062506)

Acute toxicity of the *C. procera* methanolic extract.

To evaluate the toxicity of the extract, an acute toxicity assay was performed as described in protocol 423 by the OECD.

Pathological findings not were observed in the organs during the necropsy of mice after euthanasia

Before administration of the extract, the mice were fasted for four hours. The extract was administered through an orogastric tube at a dose of 2,000 mg/kg body weight and food was withheld for an additional 2 hours.

Mice receiving the methanolic extract of *C. procera* for 14 days did not show any signs of toxicity, nor were any pathological findings observed in the organs during the necropsy of mice after euthanasia.

Females

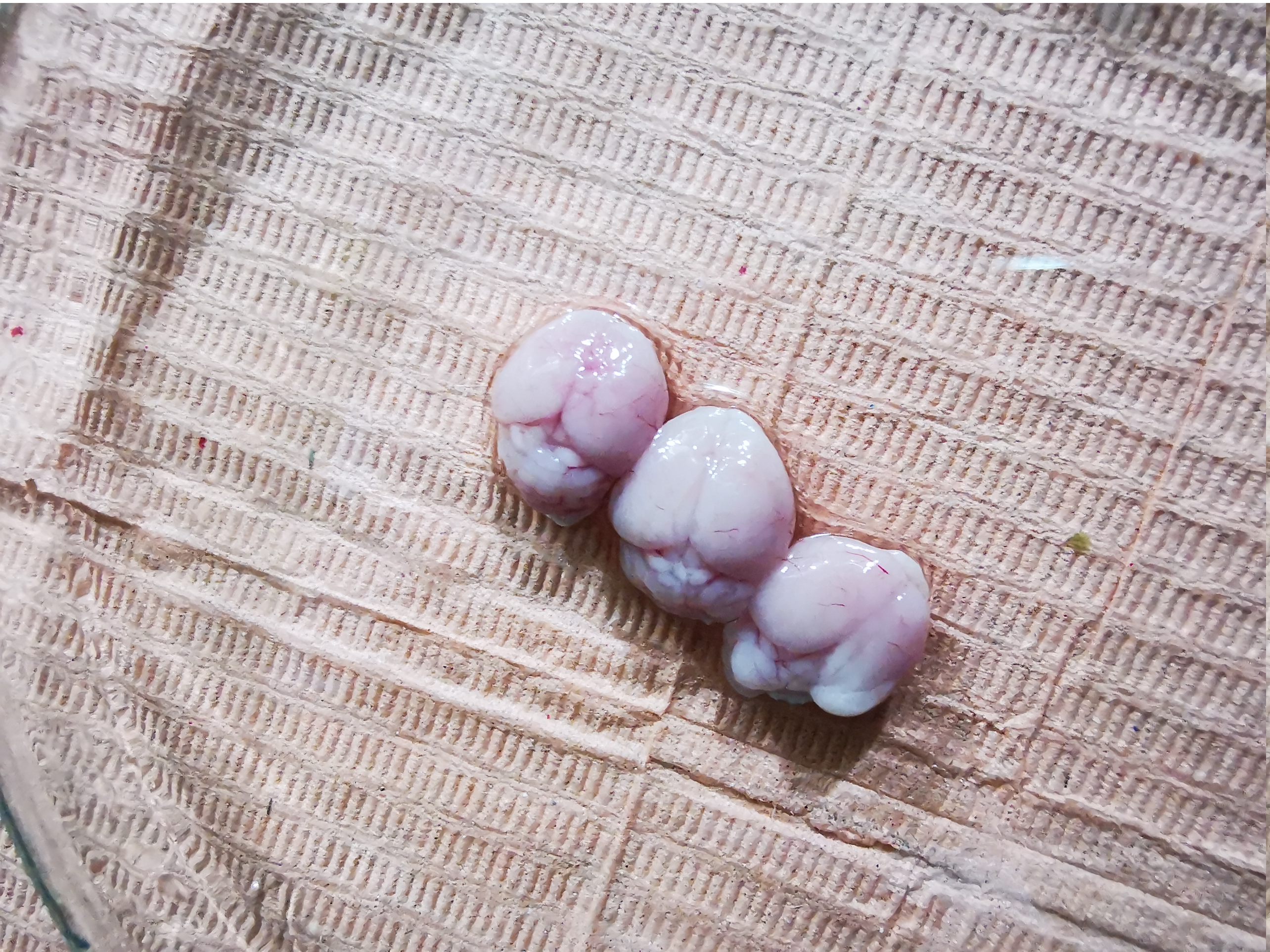

Males

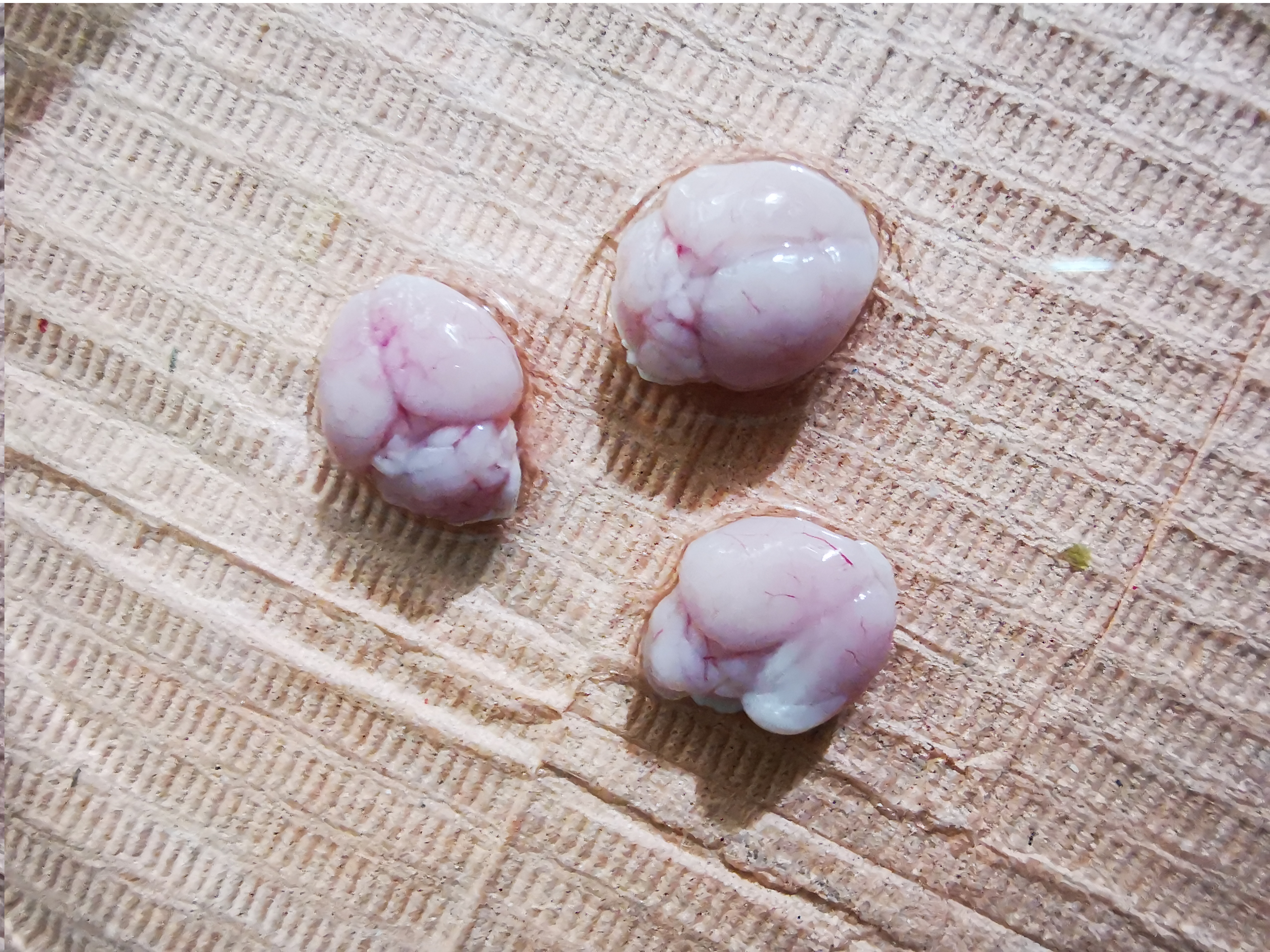

Females

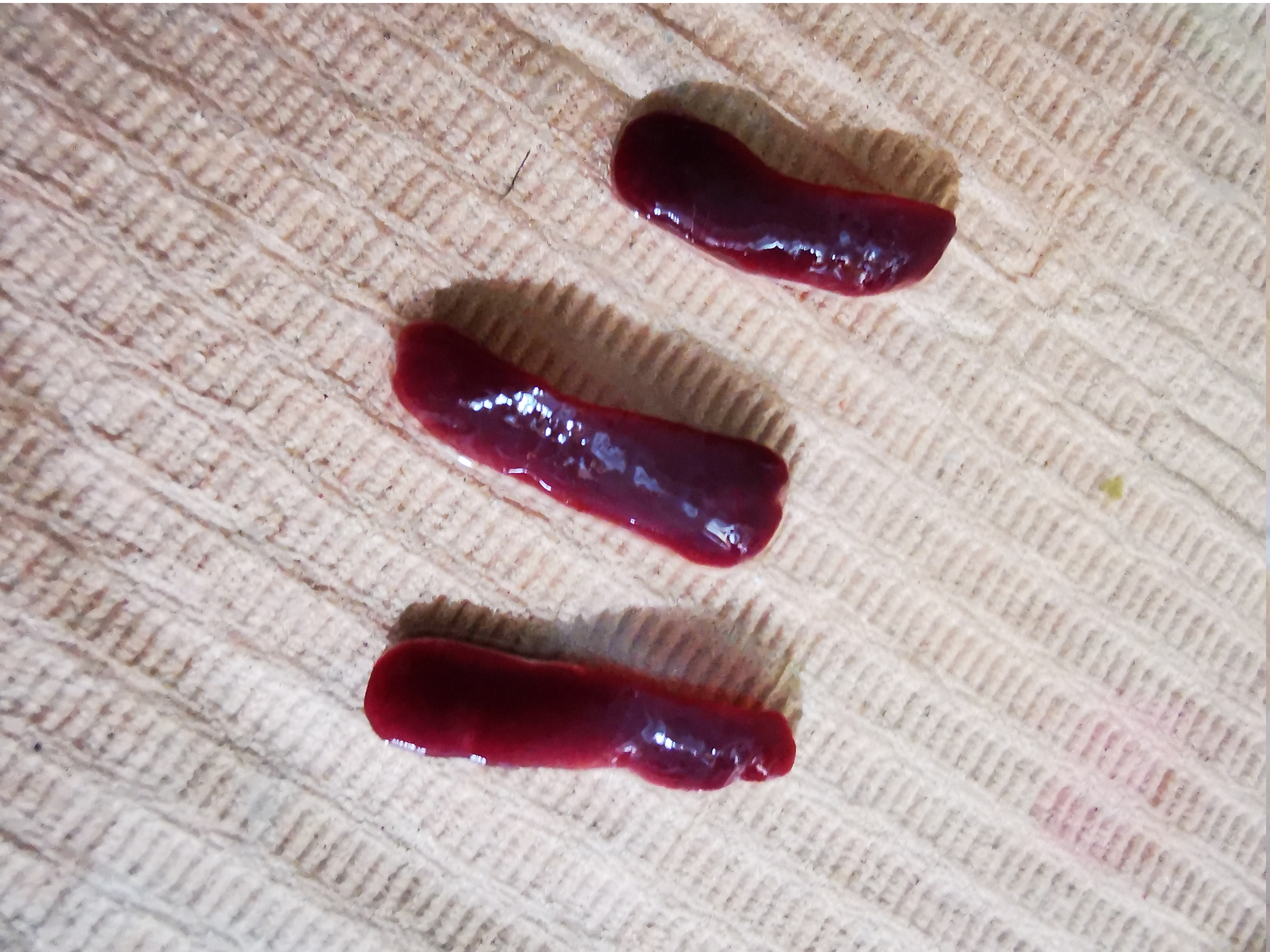

Males

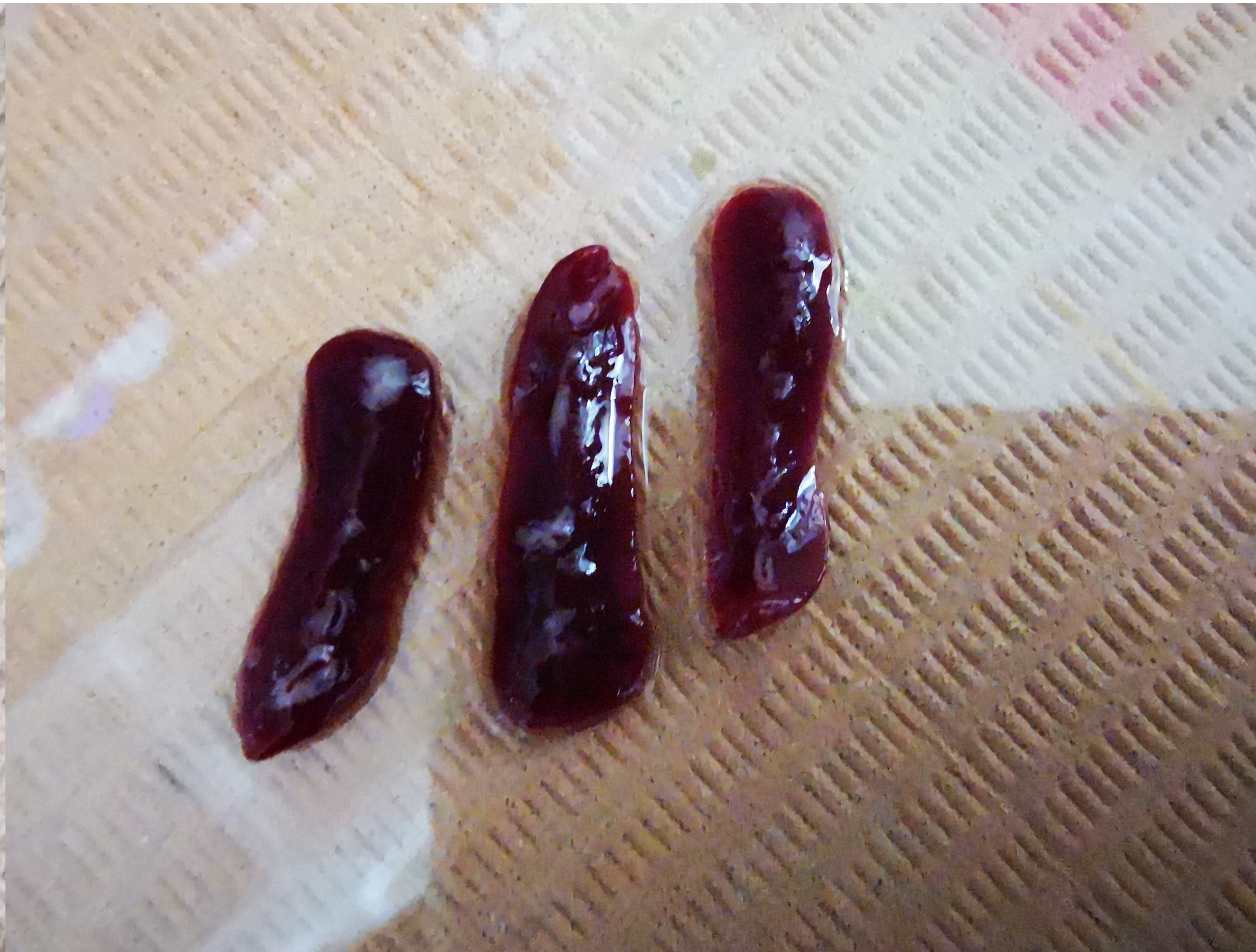

Females

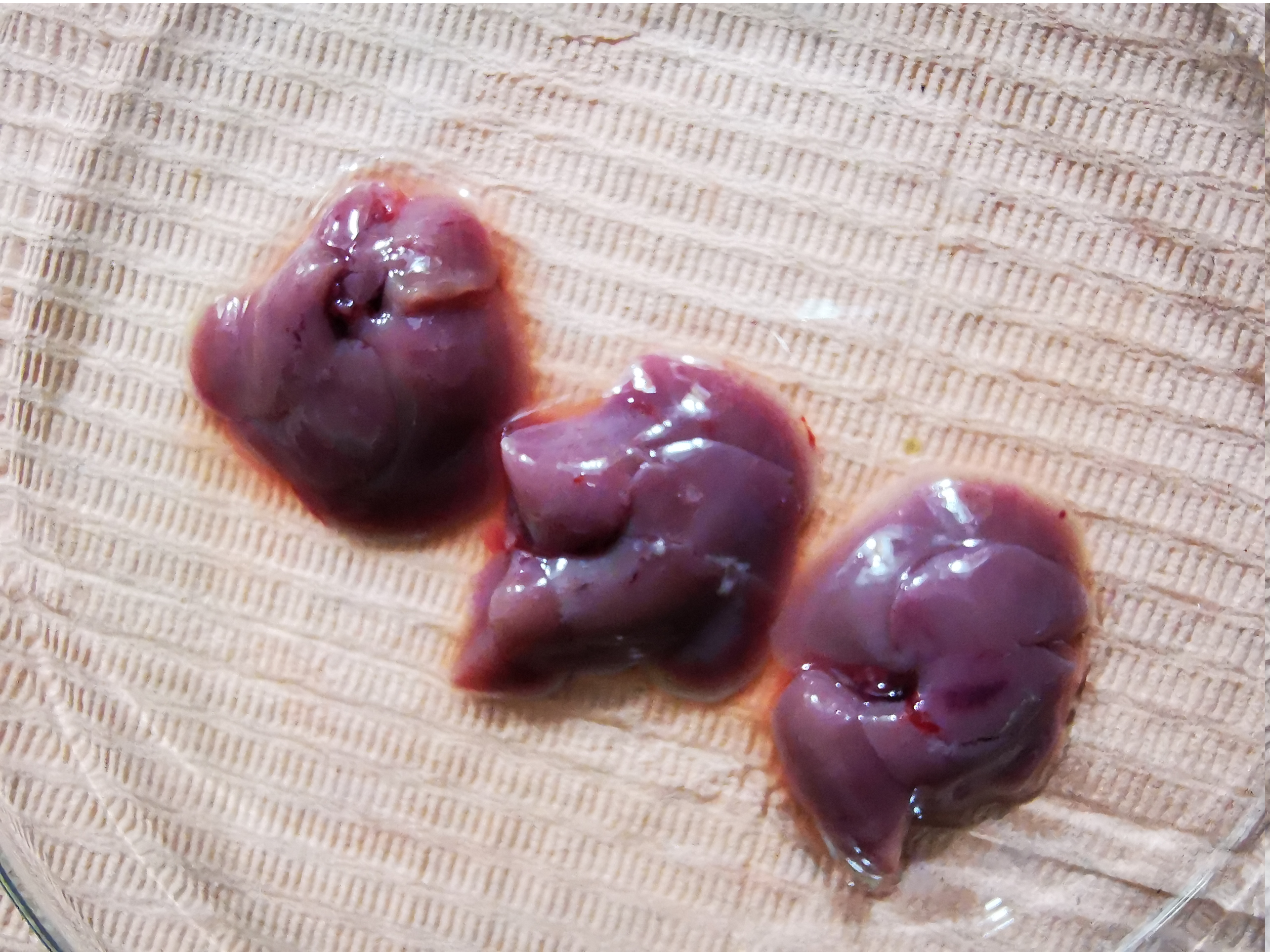

Males

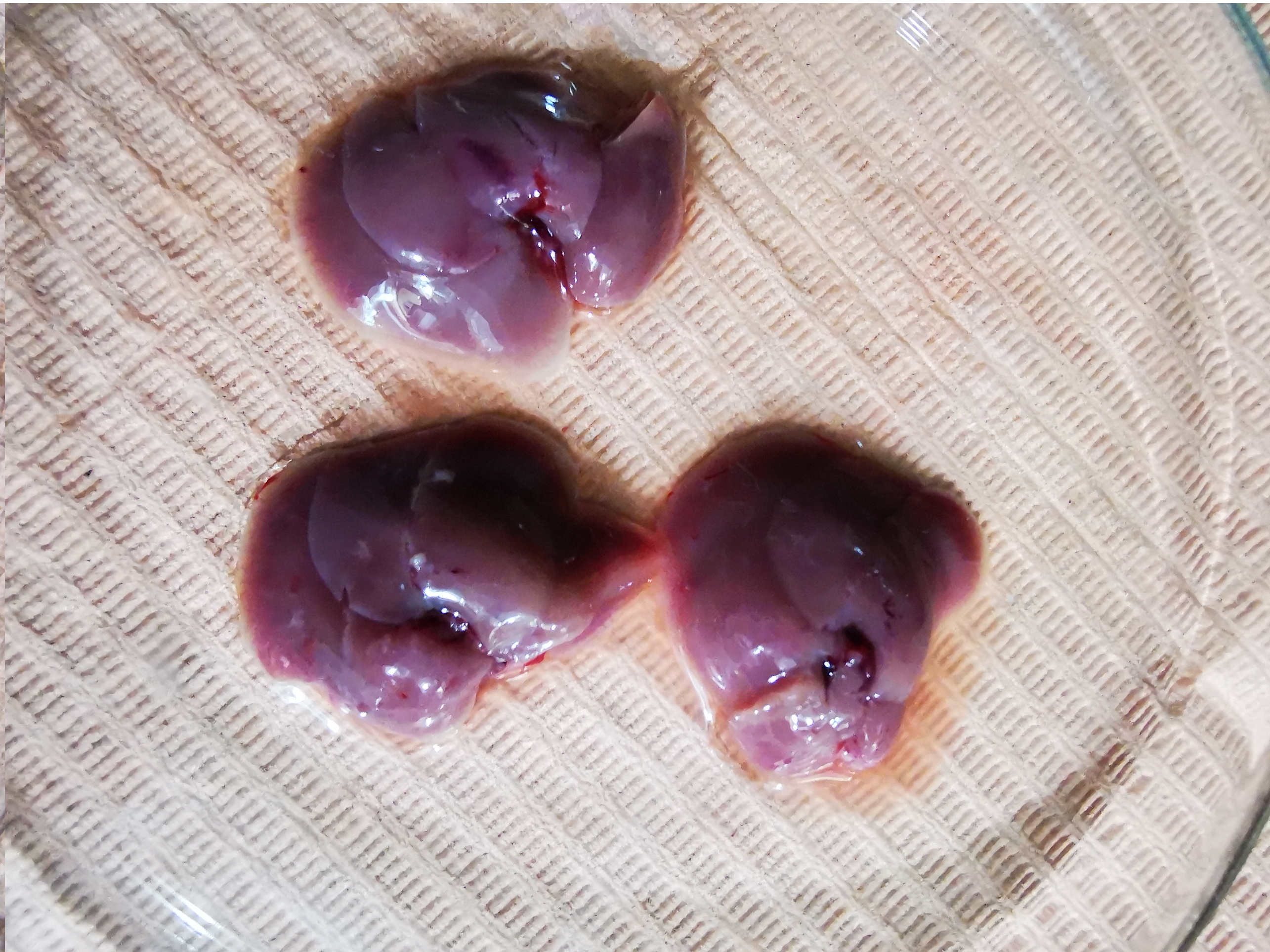

Females

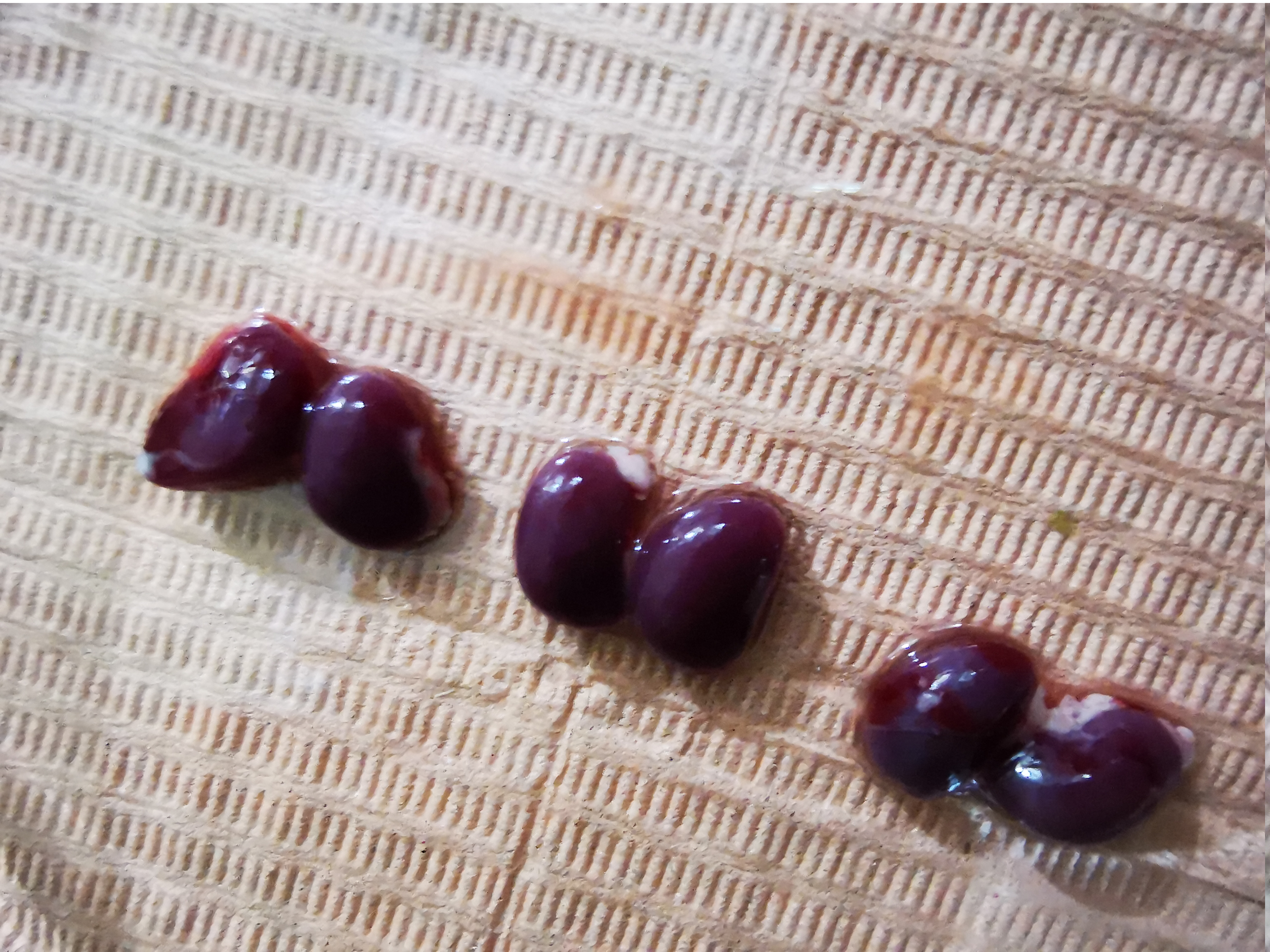

Males

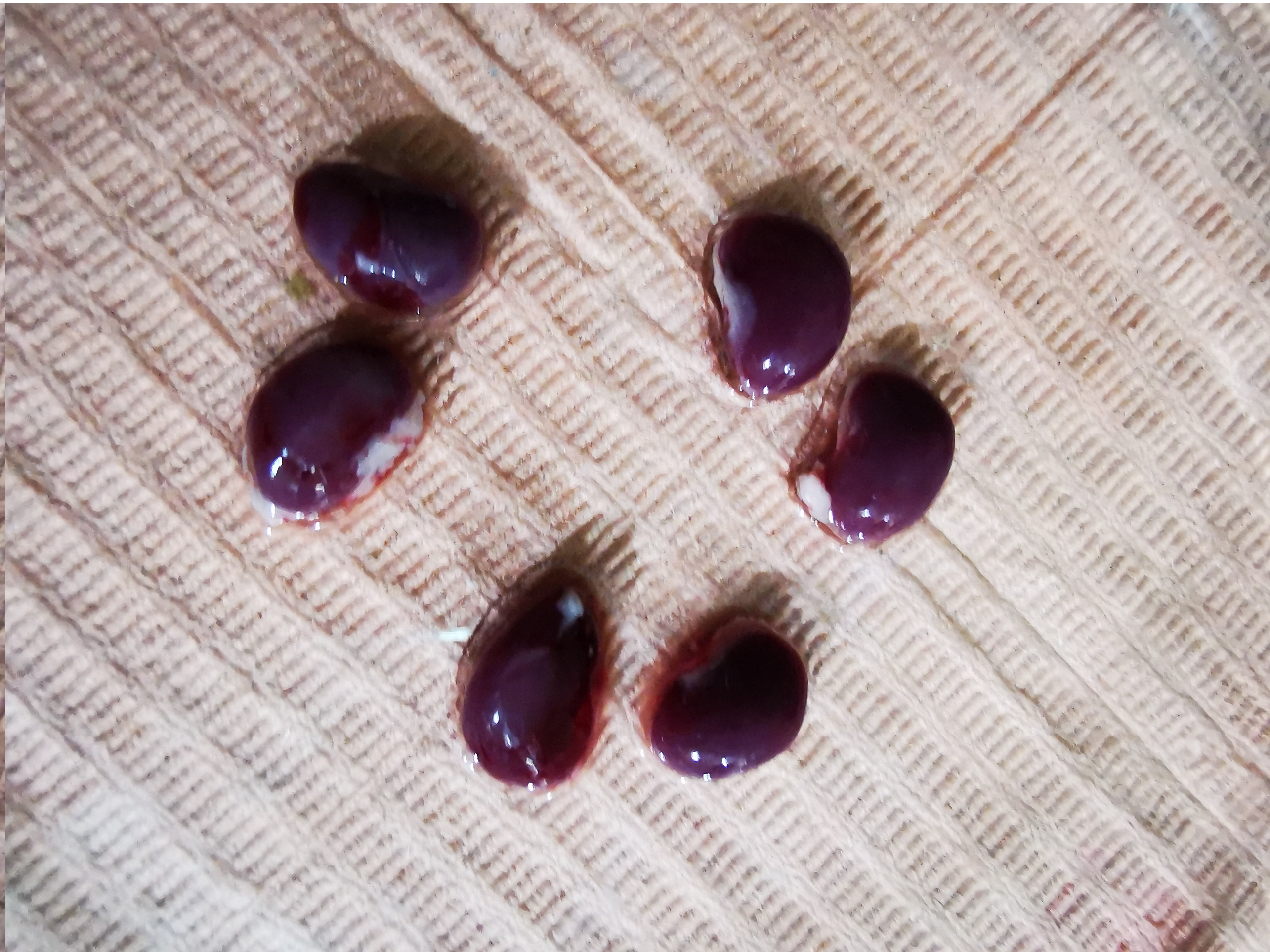

Supplement: Supplementary Materials — Reporting in vivo experiments, in which we point out the points that our study meets with the NC3Rs initiative to improve the design, analysis, and publication in animal research. The photographic record of the toxicity assessment of the extract. This toxicity assay was performed as described in protocol 423 by the OECD. Pathological findings not were observed in the organs during the necropsy of mice after euthanasia. Before the administration of the extract, the mice were fasted for four hours. The extract was administered through an orogastric tube at a dose of 2,000 mg/kg body weight, and food was withheld for an additional 2 hours. Mice receiving the methanolic extract of C. procera for 14 days did not show any signs of toxicity, nor were any pathological findings observed in the organs during the necropsy of mice after euthanasia. [file 5062506.f1.zip › Presentación1.pdf]
